# The Advantage of Low-Delta Electroencephalogram Phase Feature for Reconstructing the Center-Out Reaching Hand Movements

**DOI:** 10.3389/fnins.2019.00480

**Published:** 2019-05-15

**Authors:** Hong Zeng, Yuanzi Sun, Guozheng Xu, Changcheng Wu, Aiguo Song, Baoguo Xu, Huijun Li, Cong Hu

**Affiliations:** ^1^Jiangsu Key Lab of Remote Measurement and Control, School of Instrument Science and Engineering, Southeast University, Nanjing, China; ^2^Mechatronics and Haptics Interfaces Laboratory, Department of Mechanical Engineering, Rice University, Houston, TX, United States; ^3^College of Automation, Nanjing University of Posts and Telecommunications, Nanjing, China; ^4^College of Automation Engineering, Nanjing University of Aeronautics and Astronautics, Nanjing, China; ^5^Guangxi Key Laboratory of Automatic Detecting Technology and Instruments, Guilin University of Electronic Technology, Guilin, China

**Keywords:** electroencephalogram, instantaneous phase, multiple linear regression, Kalman filter, movement reconstruction, neuroprosthesis, brain-machine interface

## Abstract

It is an emerging frontier of research on the use of neural signals for prosthesis control, in order to restore lost function to amputees and patients after spinal cord injury. Compared to the invasive neural signal based brain-machine interface (BMI), a non-invasive alternative, i.e., the electroencephalogram (EEG)-based BMI would be more widely accepted by the patients above. Ideally, a real-time continuous neuroprosthestic control is required for practical applications. However, conventional EEG-based BMIs mainly deal with the discrete brain activity classification. Until recently, the literature has reported several attempts for achieving the real-time continuous control by reconstructing the continuous movement parameters (e.g., speed, position, etc.) from the EEG recordings, and the low-frequency band EEG is consistently reported to encode the continuous motor control information. Previous studies with executed movement tasks have extensively relied on the amplitude representation of such slow oscillations of EEG signals for building models to decode kinematic parameters. Inspired by the recent successes of instantaneous phase of low-frequency invasive brain signals in the motor control and sensory processing domains, this study examines the extension of such a slow-oscillation phase representation to the reconstructing two-dimensional hand movements, with the non-invasive EEG signals for the first time. The data for analysis are collected on five healthy subjects performing 2D hand center-out reaching along four directions in two sessions. On representative channels over the cortices encoding the execution information of reaching movements, we show that the low-delta EEG phase representation is characterized by higher signal-to-noise ratio and stronger modulation by the movement tasks, compared to the low-delta EEG amplitude representation. Furthermore, we have tested the low-delta EEG phase representation with two commonly used linear decoding models. The results demonstrate that the low-delta EEG phase based decoders lead to superior performance for 2D executed movement reconstruction to its amplitude based counterparts, as well as the other-frequency band amplitude and power based features. Thus, our study contributes to improve the movement reconstruction from EEG by introducing a new feature set based on the low-delta EEG phase patterns, and demonstrates its potential for continuous fine motion control of neuroprostheses.

## Introduction

Brain-machine interfaces (BMI) are tools that potentially enable the severely paralyzed patients after spinal cord injury (SCI) to restore lost motor ability by using the neuronal signals to control prostheses (Thomschewski et al., 2017; Lisi et al., 2018). A lot of studies have been reported in the literature, which provide discrete control for effectors by detecting and classifying discrete motor activity from the neuronal signals (Mcmullen et al., 2014; Meng et al., 2016; Yang, 2017; Zeng et al., 2017a). Nevertheless, continuous movement parameters (such as position, speed, and etc.) for the external actuator are generally demanded for realizing fine control of neuroprosthesis. In other words, the continuous BMI control, where the movement parameters are desired to be continuously reconstructed from movement-related brain activities during the (executed, attempted, or imagined) movements, would be more effective than the discrete BMI control. Such a continuous kinematics decoding approach has been successfully applied for closed-loop prosthesis control with invasively recorded brain activities, such as single/multi-unit activity (SUA/MUA) (Georgopoulos et al., 1986; Paninski et al., 2004), local potential field (LFP) (Rickert et al., 2005), electrocorticography (ECoG) (Hammer et al., 2013; Xie et al., 2017) etc., from monkeys and humans. However, such invasive BMI suffers from the possible post-surgery complications and infections, and it is also difficult to maintain stable chronic recordings. Therefore, the invasive BMI has only gained limited use among the SCI patients. Although scalp recordings such as Electroencephalogram (EEG) and magnetoencephalography (MEG) can be obtained in a non-invasive manner, it has long been believed that they lack of sufficient signal-to-noise ratio and spatial resolution to reconstruct such continuous kinematic parameters, for controlling the neuroprosthesis.

Until recently, there have been attempts gradually emerging in the research filed of continuous kinematics decoding from non-invasive scalp EEG recordings (Robinson and Vinod, 2016). The feasibility of hand movement kinematics decoding from EEG was first inspected in Bradberry et al. (2009, 2010)(Bradberry et al., 2009, 2010), during a center-out 3D hand movement experiment. They find that the hand-movement velocity information is encoded in the low-pass filtered EEG (≤2 Hz), from which the kinematic parameters are reconstructed using a linear decoding model. In Agashe et al. (2015), using a 3D reach-to-grasp experiment paradigm, the hand joint angular velocities and synergistic trajectory were decoded using low-delta band EEG (≤1 Hz). In Kim et al. (2015), the low-delta band EEG was further utilized for decoding complex 3D movement trajectories with non-linear models, in executed, observed/imagined complicated upper limb motor tasks. Researchers in Korik et al. (2016, 2018) have also shown that the low-delta band EEG is informative about the 3D hand joint trajectories in either executed or imagined arm movements, though it is not the best representation for such a 3D imagined movement decoding task according to their experimental results. As for an executed 2D center-out reaching paradigm, the low frequency EEG signal obtained with wavelet analysis has succeeded to be applied for estimating the hand kinematics adaptively (Robinson et al., 2015). The applicability of the decoding model based on delta and beta bands EEG in premotor, posterior parietal, and occipital areas for predicting the 2D hand movement velocity was demonstrated in Lv et al. (2010). The movement kinematics during the task of filling a glass of water, was studied in Heger et al. (2012) using EEG slow potentials in the delta and theta band. In a word, the above studies with executed movement tasks have consistently reported that the low-frequency EEG encodes the continuous motor control information, and the amplitude of such slow oscillations has been commonly utilized as the feature/predictor for building the linear decoding model.

While the relationship between slow oscillation EEG amplitude patterns (i.e., intensities of slow oscillations) and kinematic variables has been studied extensively, there is mounting evidence that the time-resolved signal phase patterns, characterizing the precise temporal structure for such slow oscillations, also embody the cortical motor control information. For example, it has been demonstrated that the instantaneous phase representation of the movement related cortical potential (MRCP) , an oscillation in 0.1–1 Hz delta band of EEG signal, can be utilized for the discrete detection of self-paced gait intention (Sburlea et al., 2017), and upper limb motion intention (Zeng et al., 2017b) before the movement onset. Moreover, they have shown that the phase feature based intention detector is more accurate than the amplitude based one. Lew et al. (2014) have also confirmed the power of instantaneous MRCP phase in predicting discrete movement directions of self-paced center-out arm reaching before the actual movement execution. Using the phase synchrony analysis, the authors in Jerbi et al. (2007) show that the slow (2–5 Hz) cortical oscillations in human M1 obtained with the MEG source imaging can be perfect neural correlates of hand kinematics in human. With the invasively recorded ECoG signals, it has already been discovered that the low-delta component phase is superior to its amplitude in decoding hand movement kinematics during an executed 1D continuous motor task (Hammer et al., 2013). However, it still remains unknown whether the decoding accuracy of continuous movement parameters based on the low-delta EEG phase feature is substantially higher than its amplitude feature.

To address such an issue, a 2D center-out reaching task was designed, where a right-handed subject performed continuous movements in four directions to reach a target. During the task, the EEG signals and the continuous hand movement parameters were recorded simultaneously. We firstly performed the neurophysiological analysis on the phase feature and the amplitude feature of the EEG slow oscillations on representative channels, and then compared the decoding accuracies of the low-delta EEG phase based linear decoder, the other bands EEG amplitude based counterparts, as well as the recently proposed mu/beta band power based ones by Korik et al. (2016, 2018). To the best of our knowledge, our work is the first study to extend the application of such a slow-oscillation phase representation for decoding continuous movement parameters, with the non-invasive EEG signals.

The rest of the paper is organized as follows: Section 2 describes the materials and methodology. Section 3 shows the results for the analysis performed and the experiment. Finally, we provide a detailed discussion in section 4 followed by conclusions in section 5.

## Materials and Methods

### Subjects

Five healthy subjects (all right-handed males, mean age = 22.2 ± 2.3 years old) were recruited from the campus and participated in the 2-session experiment within two separate weeks. This study was carried out in accordance with the recommendations of the Ethics Committee of Southeast University with written informed consent from all subjects. All subjects gave written informed consent in accordance with the Declaration of Helsinki. The protocol was approved by the Ethics Committee of Southeast University.

### Experimental Protocol

**Figure 1:**
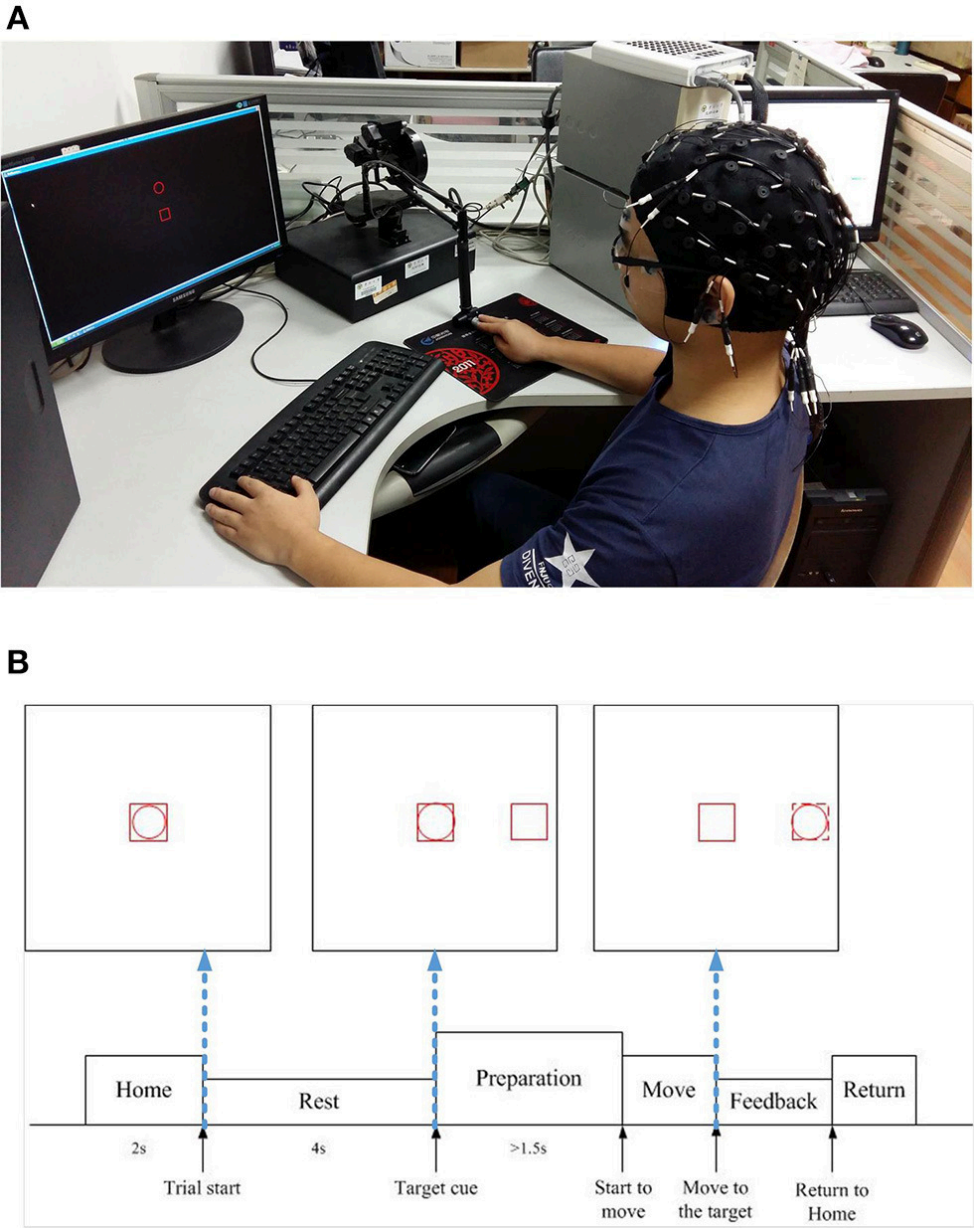
Experiment settings in our study. **(A)** Experimental setup (written informed consent was obtained from the subject for the publication of this image). **(B)** Experimental protocol timeline.

The experimental setup and the timeline in a trial are depicted in Figures 1A,B, respectively. The subject was required to sit in the chair and move the cursor (the red circle frame) from the center to one of the four orthogonal directions (up, down, left, right) in a horizontal 2D plane, by operating the haptic manipulandum (PHANTOM Premium 1.5, Sensable Technologies) with his/her right hand. In each trial, the subject first took a rest for 4 s, where the cursor (the red circle) stayed inside the red square box in the center of the screen (i.e., the “Home” position). Then a red square frame (target cue) appeared randomly in one of the four directions, which was about 10 cm away from the central on the screen. After waiting at least 1.5 s before initiating the movement, he/she moved the cursor at his/her own pace until it touched the red square frame (target cue). If the subject moved before 1.5 s (i.e., an immediate reaction), the trial was stopped, discarded from the analysis, and repeated until the subject successfully fulfilled the requirement of 1.5 s waiting period. Such a time gap design for our experiment paradigm was to avoid the visual cue evoked potentials, which might interfere with the self-paced movement related EEG signals. Subsequently, the manipulandum was moved back to the Home position for the next trial.

The experiment was conducted for two sessions for five subjects. Each session was recorded in 1 week and composed of six blocks, each consisting of 40 trials (break intervals with a duration adjusted to the need of the participants were inserted between each block), thus resulting in overall 240 trials for each subject in each week (session). It took 0.320 ± 0.126 (mean ± standard deviation) seconds on average for each subject to complete the center-out reaching movement in the experiment.

### EEG and Kinematic Recording

The EEG signals and electrooculograph (EOG) signals were simultaneously recorded with a Neuroscan SynAmps II amplifier and a 64-channel EEG cap with 10/20 montage. The electrodes impedance was kept below 5 KΩ during the recordings. Referring to the previous related studies (Bradberry et al., 2010; Robinson et al., 2015), 33 channels^1^ that distribute around frontal cortex and the parietal cortex were selected. EOG channels were placed above nasion and below the outer canthi of both eyes in order to capture horizontal and vertical EOG signals for assisting the rejection of EOG artifacts contaminated EEG trials. The EEG and EOG signal were sampled at 1000 Hz, filtered at a lower cut-off frequency of 0.05 Hz and the signal bandwidth (highest frequency) is limited at 200 Hz by the acquisition system. In order to reduce the EOG artifact contaminated EEG trials, the subjects were also required to fixate on the central square box and avoid blinking during the arm movement, but they were encouraged to blink during the period of returning to the Home position from the target. Besides, the EEG signals in a trial were carefully monitored on the Nuroscan 4.3 recordings software interface during each center-out reaching movement. Once there was a significant contamination by EOG artifacts found in the EEG recordings (e.g., EOG artifacts above 70 μV peak-peak in any channel), the trial was abandoned and the subjects were required to conduct a new movement for collecting a new trial of EEG signal.

The hand movement parameters (position and speed in X, Y axis) during the center-out reaching were recorded by the haptic manipulandum. The movement onset is defined as the time instance when the cursor (the red circle frame) exits the red square box in the center of the screen, and the end of the center-out reaching movement in a trial is defined as the time instance when the cursor hits the target square box (see Figure 1B). Then such events were sent as synchronization triggers to the EEG acquisition system via the parallel port. All the trials were then epoched from 1 s prior to the movement onset until the end of reaching movement. The datasets used and/or analyzed during the current study are available from the corresponding author on a reasonable request.

### Signal Preprocessing

To remove EOG and (electromyograph) EMG artifacts, the independent component analysis (ICA) was employed to decompose the EEG data. The criteria for identifying non-brain artifact contaminated component activations are summarized as follows: (1) Eye blinks should lie in the frontal areas with evident punctate activations. (2) Muscle activities should lie in temporal areas with a spectral peak in the band above 20 Hz. Based on these criteria, the component activations representing the EOG and EMG artifacts were removed, and the EEG data were reconstructed from the remaining component activations. The signals were further re-referenced with common average reference (CAR) to increase the signal-to-noise ratio.

Previous works (Bradberry et al., 2010; Kim et al., 2015) have extensively revealed that the low-delta EEG signals encode the motor control information, thereby a 0.1–1 Hz, zero-phase, second-order, band-pass Butterworth filter was applied. Finally, the recorded EEG signal and movement parameters were both down-sampled to 100 Hz.

### Feature Extraction and Neurophysiological Analysis

The preprocessed EEG sequence is then used to define a predictor set from which the estimator for the movement parameter information will be built. In this study, we will derive the corresponding phase sequence of the preprocessed EEG sequence for building the predictor set. For such a narrow-band signal, i.e., the temporal amplitude sequence of the low-pass-filtered EEG, its instantaneous phase at each time point can be obtained with the analytic representation:

(1) $$\begin{array}{r} z\left(t\right)=s\left(t\right)+jHT\left(s\left(t\right)\right) \end{array}$$

where ***HT***(*s*(*t*)) is the Hilbert transformation of the sequence *s*(*t*), defined as

(2) $$\begin{array}{r} HT\left(s\left(t\right)\right)=s\left(t\right)*\frac{1}{\pi t} \end{array}$$

Here ^*^ denotes the convolution. The instantaneous phase sequence φ(*t*) is defined as the angle of such an analytical signal:

(3) $$\begin{array}{r} \phi \left(t\right)=\begin{array}{cc} \arctan & \frac{HT\left(s\left(t\right)\right)}{s\left(t\right)} \end{array} \end{array}$$

We further conducted the neurophysiological analysis at two levels. First, a spatio-temporal analysis was performed to investigate two kinds of features at the scalp level:

(1) The amplitude features of the signal filtered in 0.1–1 Hz;

(2) The instantaneous phase features of the above signal obtained with Equation (3).

Second, we conducted statistical analyses to assess two metrics of such two types of features from several selected representative channels (the method for selecting the representative channel will be specified later) over the frontal and parietal cortices [such two cortices are extensively reported to carry necessary information for decoding planning and execution of reaching movements in the literature (Lew et al., 2014; Robinson et al., 2015)]. The two metrics for characterizing these two types of features are illustrated as follows:

(1) The time-resolved effect size for each type of feature in the movement sub-interval ^2^ (0–0.2 s relative to the movement onset) relative to the rest (baseline) interval (between −0.2 and −0.1 s relative to the movement onset), which actually measures the signal-to-noise ratio of the feature. In specific, we firstly computed the grand average of the two features over trials across subjects and sessions. Nextly, the mean and standard deviation of the baseline were calculated. Finally, the effect size for each type of feature was obtained by subtracting the grand average baseline from the grand average activity of the movement sub-interval, and being divided by the standard deviation of the baseline.

(2) The absolute Pearson correlation coefficient (CC) which measures the strength of linear correlation relationship between the feature and recorded movement speed in the movement duration. The weakest linear relationship is indicated by 0, whereas the strongest one is represented by 1.

Since the frontal and parietal areas are generally believed to encode the planning and execution information of reaching movements, we restricted the statistical analysis and visualization only to the features from the representative channels in frontal and parietal areas, respectively. The representative channels were screened using the electrode activation index (AI) (Benz et al., 2012), which measures the average level of change in low-delta EEG features between baseline and movement. In specific, we utilized the cross-correlation coefficient to compare the amplitude feature and the phase feature, between the baseline state (rest, *r*) and active state (hand movement, *m*):

(4) $$\begin{array}{r} F=\left|\frac{{\left(m-r\right)}^{3}}{|m-r|\sigma_{m\cup r}^{2}}\frac{N_{m}N_{r}}{N_{m\cup r}^{2}}\right| \end{array}$$

where *r* represents the average feature value during rest, *m* denotes the average feature value across all hand movements, σ is the variance of the feature across the movements and rest states, *N*_*m*_ and *N*_*r*_ denote the number of incidences of each state, respectively. The cross-correlation coefficient of each channel was calculated with all the trials from five subjects and his/her two sessions, using both the amplitude and phase feature, respectively. Then the electrode AI was determined for each channel as follows:

(5) $$\begin{array}{r} \text{AI}=\text{max}\left\{{\text{F}}_{\text{amplitude}},{\text{F}}_{\text{phase}}\right\}, \end{array}$$

i.e., the amplitude or phase derived cross-correlation coefficient that shows a greater change between the rest and movement states for all the trials. For each of the four directions, we selected a channel with the highest AI from the frontal cortex and the parietal cortex, respectively. In this manner, we obtained the most representative channels from the four-direction movement-related cortex.

Subsequently, we performed the statistical analysis on the above two metrics of the amplitude and phase features from these representative channels. The pairwise Wilcoxon signed rank test was conducted on the absolute values of the two metrics, where the statistical significances for the effective size analysis was confined to the movement sub-interval (0–0.2 s).

### Decoding Models

For the decoding, the linear decoding methods were commonly employed in previous studies for decoding continuous movement, such as multiple linear regression (MLR) (Bradberry et al., 2010) and Kalman filter (KF) (Wu et al., 2006; Pistohl et al., 2008). Since our study aims at investigating whether the low-delta EEG phase feature based linear decoding models could substantially improve on the amplitude feature based ones, we have employed the commonly used MLR and KF for the continuous movement decoding.

In our study, we define the movement parameters as

(6) $$\begin{array}{r} \text{Y}=\left\{s_{x},s_{y},d_{x},d_{y}\right\}\in {\mathbb{R}}^{4\times \left(n_{1}+n_{2}+\cdots n_{N}\right)} \end{array}$$

where *n*_i_ is the number of instances in the i-th trial (i = 1,…, N). Thus, the absolute speed and position can be calculated as: $s=\sqrt{s_{x}^{2}+{s_{y}}^{2}}$ and $d=\sqrt{d_{x}^{2}+{d_{y}}^{2}}$. The i-th trial recorded brain activity is denoted by $\text{E}\in {\mathbb{R}}^{C\times n_{i}}$, where *C* is the number of channels recorded. Next, we derive the low-delta EEG amplitude representation $\text{A}\in {\mathbb{R}}^{C\times n_{i}}$ and the low-delta EEG instantaneous phase representation $\text{P}\in {\mathbb{R}}^{C\times n_{i}}$for the single trial brain activity **E**. In such two representations, signals from each channel are further normalized to unit Euclidean length. The predictor set $X\in {\mathbb{R}}^{L\times \left(n_{1}+n_{2}+\cdots n_{N}\right)}$ where *L* = *C* · θ is then defined as

(7) $$\begin{array}{r} \left\{\begin{array}{ccc} {\text{x}}_{\tau c}\in {\mathbb{R}}^{1\times \left(n_{1}+n_{2}+\cdots n_{N}\right)}, & \tau =1,\ldots ,\theta & c=1,\ldots ,C \end{array}\right\}, \end{array}$$

where **x**_τ*c*_ represents the feature (amplitude or phase) from channel *c* and delayed by τ−1 for the N trials of the original low-delta EEG signal. In Equation (7), θ is the embedding dimension that equals to the number of time delays plus one, and it actually specifies that there are one sample at time instance *k* and (θ−1) samples prior to instance *k* from each channel needed for estimating the movement parameters at time instance *k*. In our analysis, the constants in the Equation (7) are set to *C* = 33 and θ = 11, respectively. In the rest of this paper, the predictor at time instance *k* will be denoted by **X**_*k*_, i.e., **x**_τ*c*_(*k*), and the movement parameter at instance *k* is **Y**_*k*_.

We have firstly employed the MLR, which fits the recorded kinematic parameters over multiple regression variables by a linear fitting strategy (Wu et al., 2006). The linear regression problem can be written as follows:

(8) $$\begin{array}{r} {\hat{\text{Y}}}_{\text{MLR}}\left(k\right)\text{=}\overset{\theta }{\underset{\tau =1}{\sum }}\overset{C}{\underset{c=1}{\sum }}\alpha_{\tau c}{\text{x}}_{\tau c}\left(k\right) \end{array}$$

Here, ${\hat{\text{Y}}}_{\text{MLR}}\left(k\right)$ is the estimated value of **Y**(*k*), α_τ*c*_ denotes the regression weight, **x**_τ*c*_(*k*) denotes the predictor at time instance *k* from channel *c* with time lag τ − 1. The regression equation in (8) can be rewritten in the following form:

(9) $$\begin{array}{r} {\hat{\text{Y}}}_{\text{MLR}}\text{=}\alpha \cdot \text{X} \end{array}$$

where an extra column of ones will be included to induce the bias in the regression model and thus **α** ∈ ℝ^4×(*L*+1)^. Such a problem can be solved by the least-square estimation.

The Kalman filter (KF) has been widely applied in decoding kinematic parameters from invasive and non-invasive neuronal activities in previous BCI studies (Wu et al., 2006; Pistohl et al., 2008). KF predicts parameters of interest from inaccurate and uncertain observations. In specific, it minimizes the mean square error of the estimated parameters in the presence of Gaussian noises with unknown mean and variances. In KF, a discrete-time linear dynamical system is modeled where the state of the system at any time instance is further defined by a linear model. A generative model will be established in the KF algorithm, assuming that the measured output (the predictor set at time instance *k*) is linearly related to the state (movement parameter at instance *k*). The generative model is defined as

(10) $$\begin{array}{r} {\text{X}}_{k}=H_{K}{\text{Y}}_{k}+q_{k}, \end{array}$$

Here *H*_*K*_ is the matrix that linearly relates the predictor with the state of the system (i.e., movement parameters). The noise *q*_*k*_ in the observations is zero mean and normally distributed. *q*_*k*_ ~ *N*(0, *Q*_*k*_), where *Q*_*k*_ is the noise covariance matrix. The Kalman filter assumes the state at time *k* + 1 is evolved from the state at *k* according to the system model.

(11) $$\begin{array}{r} {\text{Y}}_{k+1}=A_{k}{\text{Y}}_{k}+w_{k}, \end{array}$$

Here *A*_*k*_ is the coefficient matrix and the noise *w*_*k*_ ~ *N*(0, *W*_*k*_), *W*_*k*_ is the noise covariance matrix. The KF method will predict the hand movement state **Y**_*k*+1_ from **Y**_*k*_ by following Equation (11). We make assumption that *H*_*k*_, *Q*_*k*_, *A*_*k*_, *W*_*k*_ are constant and estimated from the training data using least square estimation method.

The reconstruction of the hand movement parameters by the KF model consists of two steps. In the first step, the system model estimates the hand movement parameters at time *k* + 1 from the state at time *k*. In the second step, these estimates are updated using a weighted average, with more weight being given to estimates with a higher certainty, once the outcome of the next measurement is observed.

### Decoding Performance Evaluation

#### Evaluation Against the Decoding Models With the Low-Delta Amplitude Feature

Since the low-delta EEG signal amplitude feature has been extensively used when building the movement decoding models in the literature (Bradberry et al., 2010; Agashe et al., 2015; Robinson et al., 2015), it is interesting to firstly assess whether phase feature of the low-delta EEG could improve up on continuous kinematic parameters decoding based on the low-delta EEG amplitude feature. To this end, we constructed linear decoders (MLR and KF) based on two sets of features: (1) Amplitude model, the decoding model (MLR/KF) based on the amplitude feature of low-delta band EEG signal. (2) Phase model, the decoding model (MLR/KF) based on the instantaneous phase feature of low-delta band EEG signal.

For each subject, since each session includes six blocks (each consisting of 40 trials), thereby the data sets in each session were naturally split into six-folds. The decoding performance was then assessed by six-fold cross-validation (CV) for data of each subject and each session, where five-folds for model building and the remaining fold as a test set for the model's kinematic parameters reconstruction. This procedure was repeated six times, such that each fold was used as test set exactly once. The decoding performance was evaluated with the correlation coefficients between the estimated (predicted) and the ground-truth kinematic recordings obtained from all test folds. We conducted the statistical analysis on the performance difference between the Phase model and the Amplitude model, using the pairwise Wilcoxon signed rank test.

#### Evaluation Against the Decoding Models With Other-Band Power and Amplitude Features

Recently, authors in Korik et al. (2016, 2018) have advocated the utilization of the time-resolved power feature extracted from other frequency bands (e.g., the mu and beta bands, etc.) EEG signals for decoding the 3D executed or imagined movement trajectories. They have shown that the bandpower feature is also an effective alternative to the slow-oscillation EEG signal amplitude feature. In this regard, we have also compared the performance of such band-pass filtered EEG power features with that of the slow-oscillation EEG phase feature. In specific, as in Korik et al. (2018), we applied the 8th order zero-phase band-pass Butterworth filters on the artifacts removed EEG in the high delta (1–4 Hz), theta (4–8 Hz), mu (8–12 Hz), and beta (12–30 Hz) bands, following a down-sampling to 100 Hz. The time-resolved bandpower was obtained by averaging the square values of the band-pass filtered EEG signals within a 500 ms-long sliding window in a step of 10 ms. Then the performance of the high delta (1–4 Hz), theta (4–8 Hz), mu (8–12 Hz), and beta (12–30 Hz) bandpower feature based MLR/KF decoding models was evaluated with the same six-fold cross-validation procedure. For completeness, the six-fold cross-validation decoding performance with the amplitude representation of those bands was reported as well. However, the phase features for other band EEG were not used to continuously predict the movement trajectory (<4 Hz), because the instantaneous phase varies faster than the relatively slow time course of the movement trajectory.

The subject-session-specific CV performance was obtained by averaging the six correlation coefficients from the six-fold cross-validation procedure for each subject and each session. We took the mean value of the two axes (X and Y) as the final CV performance to report as in Fernandez-Vargas et al. (2016) and Li et al. (2018). The statistical significance analysis on the performance differences (among the low-delta EEG phase based model, the other-band EEG power based one and the amplitude based one) was further conducted with the one-way ANOVA using the Tukey's honestly significant difference correction procedure for multiple comparisons. The normality assumption of the ANOVA was validated by the Jarque-Bera test.

#### Evaluation Against the Chance-Level Decoding Models

To ensure the validity of the proposed Phase model for reconstructing the hand movement trajectory, it was further tested against the chance level of the reconstruction. The chance-level reconstruction was empirically obtained by first shuffling the original data (i.e., disorganizing the correspondence between original EEG signals and position/speed profiles) and then applying the phase feature based MLR/KF decoding model on the shuffled data. We repeated the shuffling process *N* = 20 times per subject and per session to reduce the chance effects due to the randomness of the process, the six-fold CV evaluation was still adopted after each shuffling process (with *N* = 10% of the number of trials used for training). The chance level results were tested against those with authentic models, using the Wilcoxon rank sum test.

## Results

### Neurophysiological Analysis

**Figure 2:**
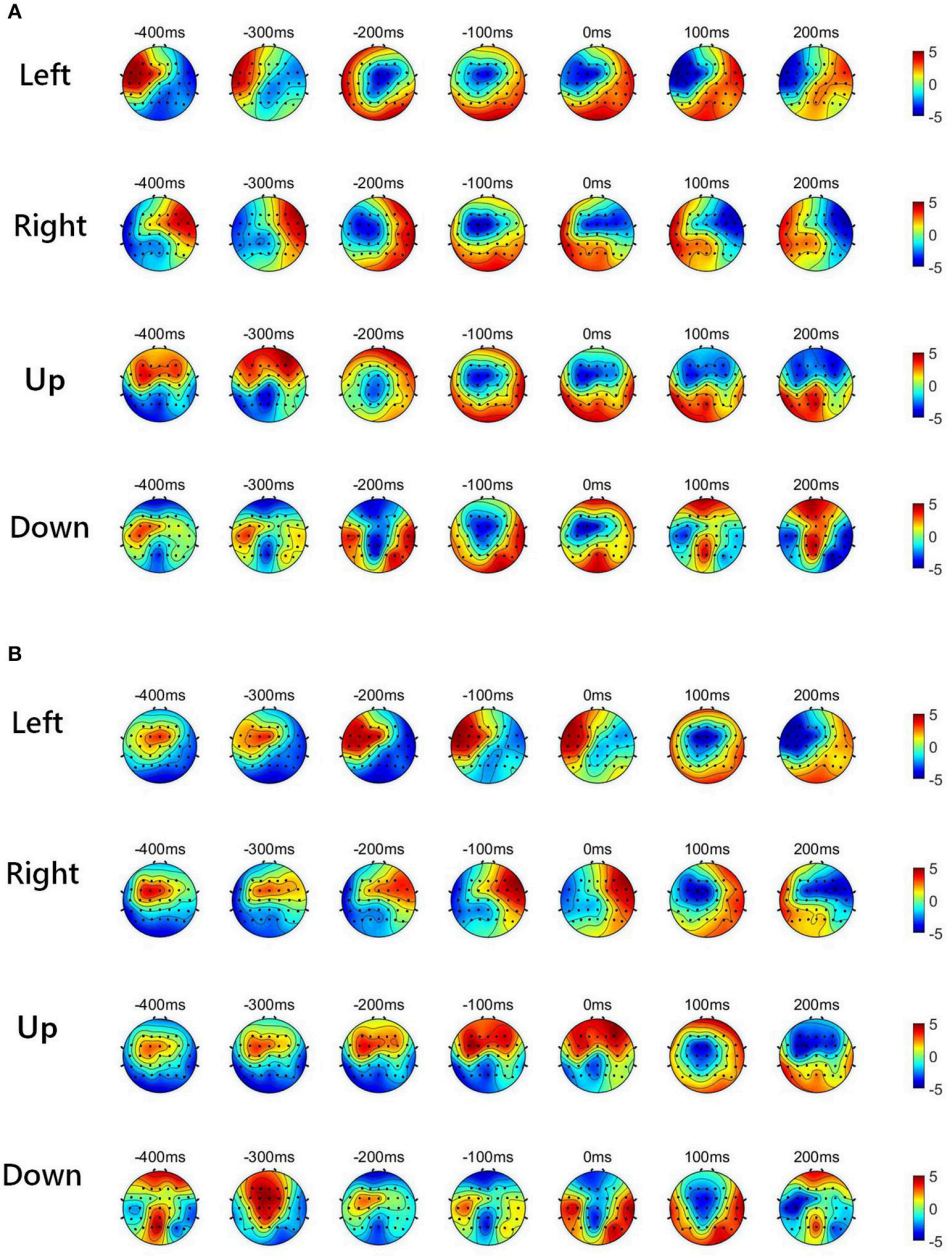
The time-resolved topographic maps of low-delta EEG features during the four-direction movements over subjects and sessions. **(A)** The grand average of low-delta EEG amplitude features. **(B)** The grand average of low-delta phase features.

**Figure 3:**
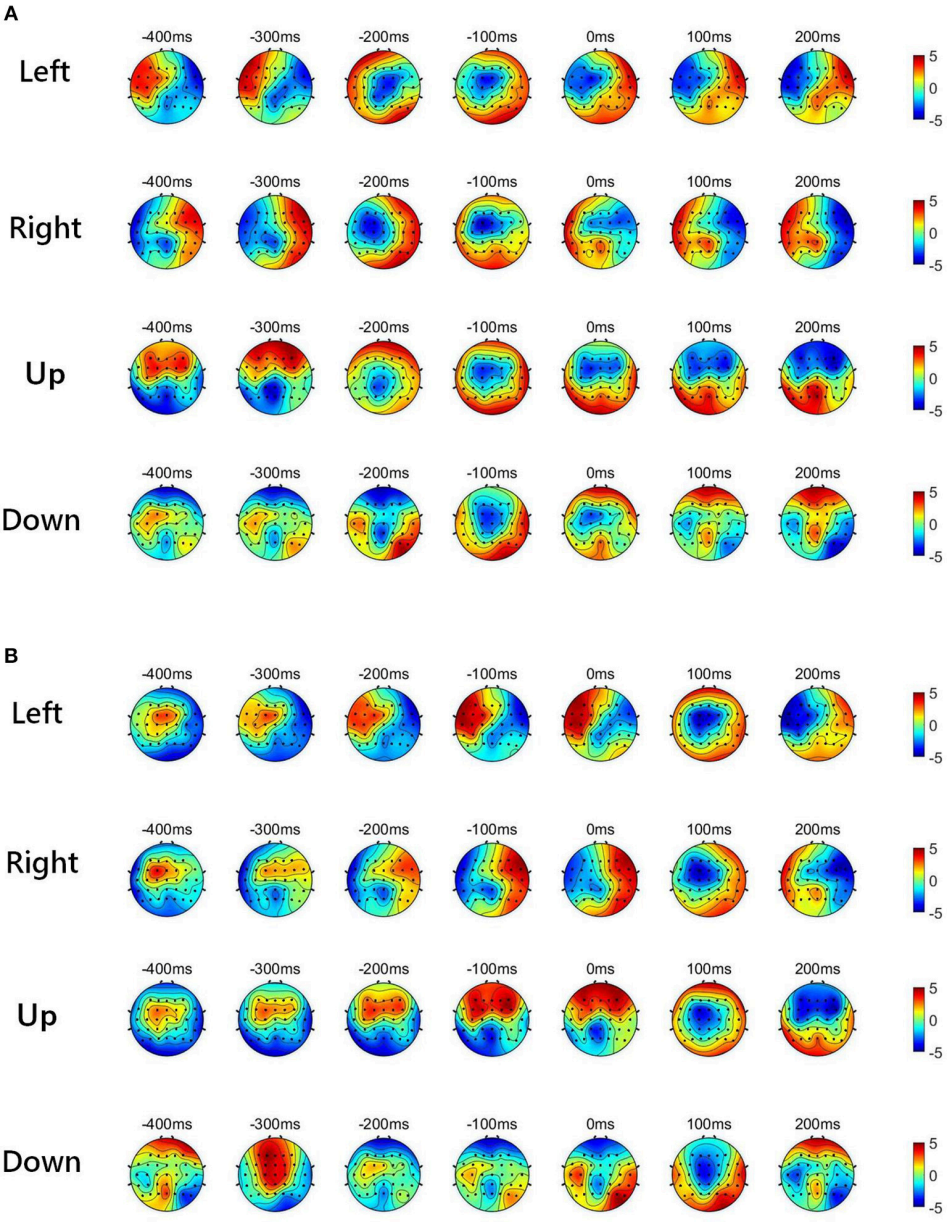
The time-resolved topographical maps of the low-delta EEG features during the four-direction movements from a representative subject and session (1st trial in the 2nd session of subject 5). **(A)** The low-delta EEG amplitude features. **(B)** The low-delta EEG phase features.

Figure 2 shows the low-delta EEG amplitude and phase features in grand average over subjects and sessions, covering the pre-movement interval [−0.4, 0] s, and the movement sub-interval [0, 0.2] s. Since we find that the topographical scalp distribution of the low-delta EEG correlates for hand movement demonstrates different patterns for the four movement directions, the topographic maps of low-delta EEG features during the four-direction movements are depicted separately. Moreover, before calculating the grand average, the amplitude feature, and the instantaneous phase feature from each channel were z-scored. From the time-resolved topographic maps of low-delta EEG amplitude features in Figure 2, we can observe that the spatio-temporal grand average of the amplitude patterns have demonstrated a smooth variation before and after the movement onset (0 s). By contrast, for the phase patterns shown in Figure 2, although the topographical scalp distribution of the phase patterns also shows a smooth change during the pre-movement stage, it has exhibited significant changes since the movement onset. In particular, the time-resolved topographical maps of the low-delta amplitude and phase features from a representative subject and trial in a session (the 1st trial in the 2nd session of subject 5) are shown in Figure 3. It can be observed that the trend of such representative spatio-temporal patterns is generally consistent with that of the grand average ones.

**Figure 4:**
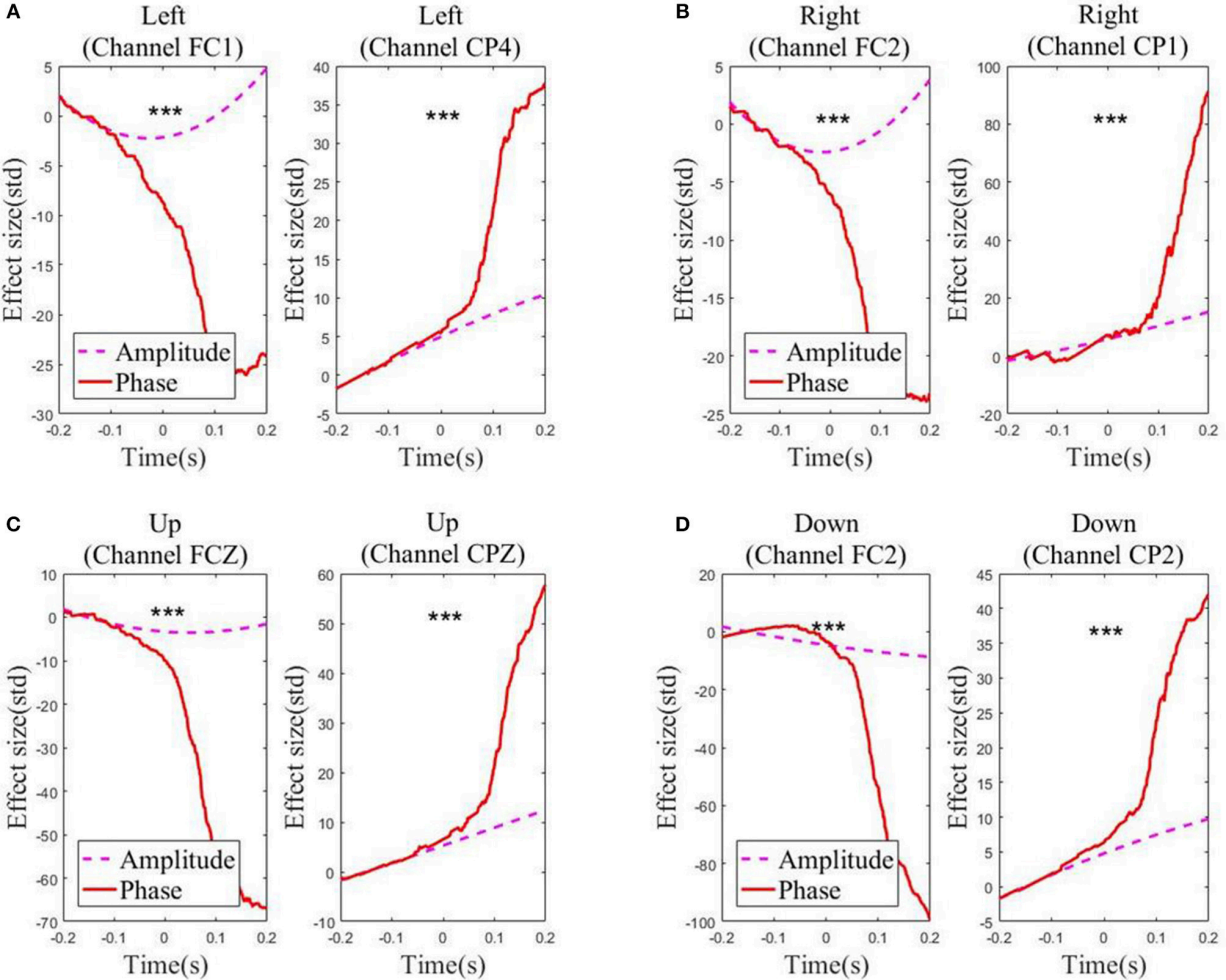
Effect sizes for the low-delta EEG amplitude feature and phase feature quantified in standard deviation units in the representative channels for the grand average across subjects and sessions along the four directions: **(A)** Left. **(B)** Right. **(C)** Up. **(D)** Down. Those channels, which exhibit statistical significant differences (signed rank test *p* << 0.01) in effect size for the amplitude feature and the phase feature during the movement sub-interval (0–0.2 s), are marked by ^***^.

For each of the four movement directions, the selected channels with the highest electrode AI over the frontal and parietal cortices, respectively, are shown in Figure 4. Figure 4 presents the effective size for each type of feature from these representative channels. In the movement sub-interval [0, 0.2] s, it can be easily observed that the phase feature generally has a much larger effective size than the amplitude feature, relative to the baseline interval [−0.2, −0.1] s. We further evaluated the statistical significance between the absolute value of the effective size of the two representations (phase, amplitude) in the movement sub-interval [0, 0.2] s. The differences between phase and amplitude are indeed found to be statistically significant (*p*-value << 0.01 on all representative channels). Such findings indicate that on these representative channels, the signal-to-noise ratio of the phase feature is substantially higher than that of the amplitude feature in the movement interval.

**Figure 5:**
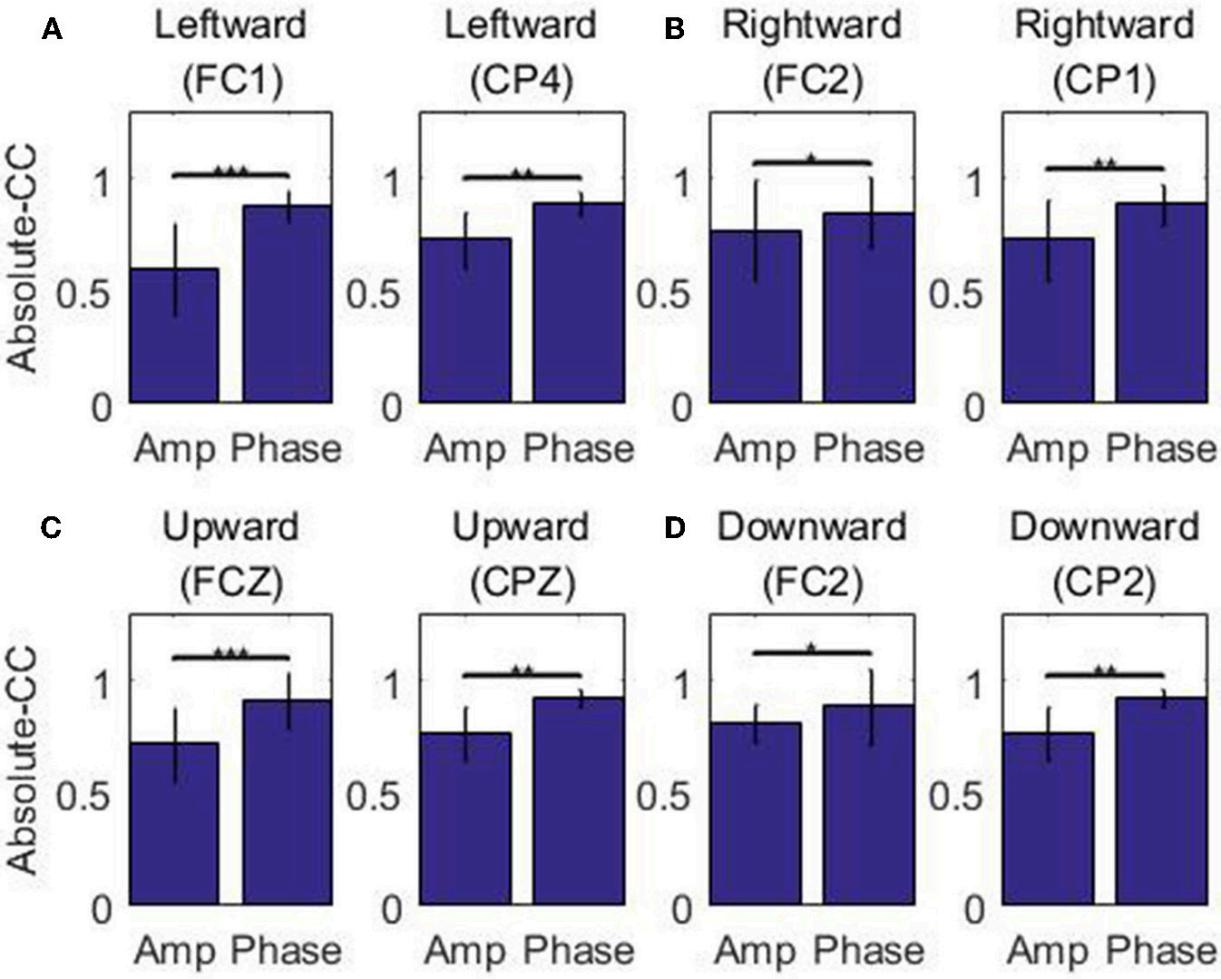
The absolute correlation coefficients between the speed and the low-delta EEG amplitude/phase feature in the representative channels across subjects and sessions along the four directions: **(A)** Left. **(B)** Right. **(C)** Up. **(D)** Down. The results demonstrating statistical significant differences are marked by ^***^ (signed rank test *p* < 0.001), ^**^ (signed rank test 0.001 ≤ *p* < 0.01) and ^*^ (signed rank test 0.01 ≤ *p* < 0.05).

**Figure 6:**
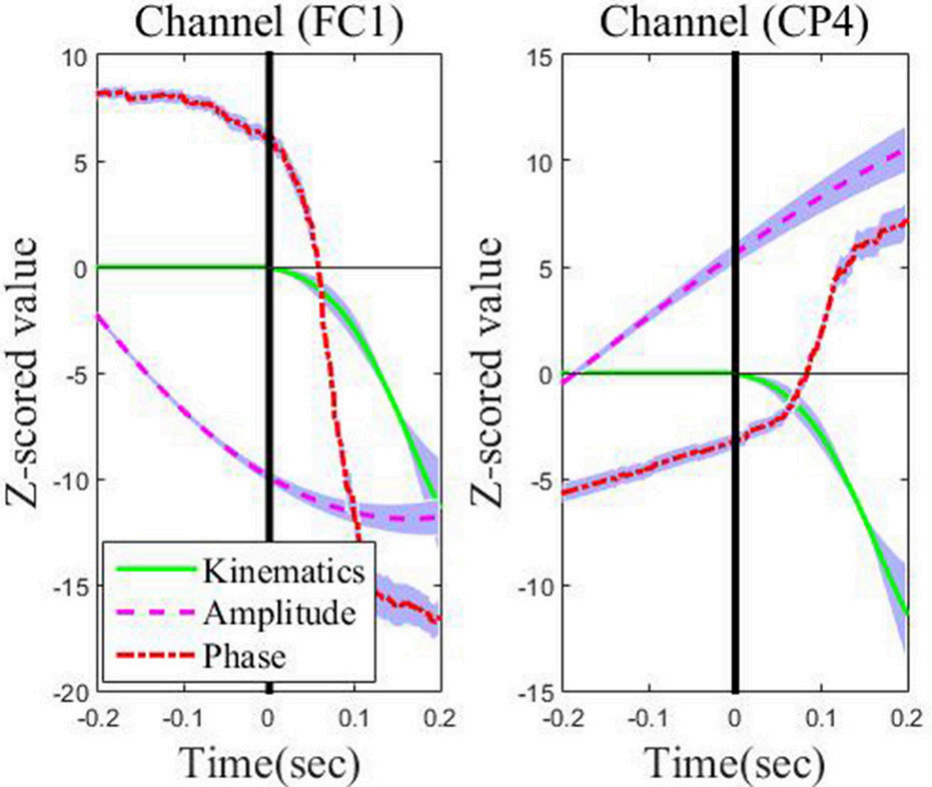
The across-subject and across-session time course of low-delta EEG phase features and amplitude features in the representative channels (FC1 and CP4) preceding movement onset (the vertical line on 0 s) and during the leftward movement. The thick lines denote the average patterns, and the range of standard deviation is plotted with a shaded background in blue.

Figure 5 depicts the absolute CC for each type of feature from these representative channels in the movement interval. From Figure 5, we can observe that the average absolute Pearson correlation coefficient with phase feature is substantially higher than that with the amplitude feature. Moreover, such differences are statistically significant (*p* < 0.05). These results clearly identify that the temporal profile of the phase features from these representative channels in the movement interval are more linearly correlated with the recorded kinematics data than the amplitude features. As an example, the z-scored values of the phase and amplitude (average and standard deviation) on the FC1 and CP4 channels and the z-scored speed (average and standard deviation) recording during the leftward movement across subjects and sessions are shown in Figure 6. Clearly, the phase feature is better aligned with the movement parameters. Moreover, it also demonstrates smaller variations (thus is more stable) across trials than the amplitude one around the motion onset, reflecting the entrainment of the oscillatory dynamics by the motion event. Such observations suggest that on these representative channels, the phase pattern is strongly modulated by the movement task, which may carry more continuous movement information than the amplitude one.

**Figure 7:**
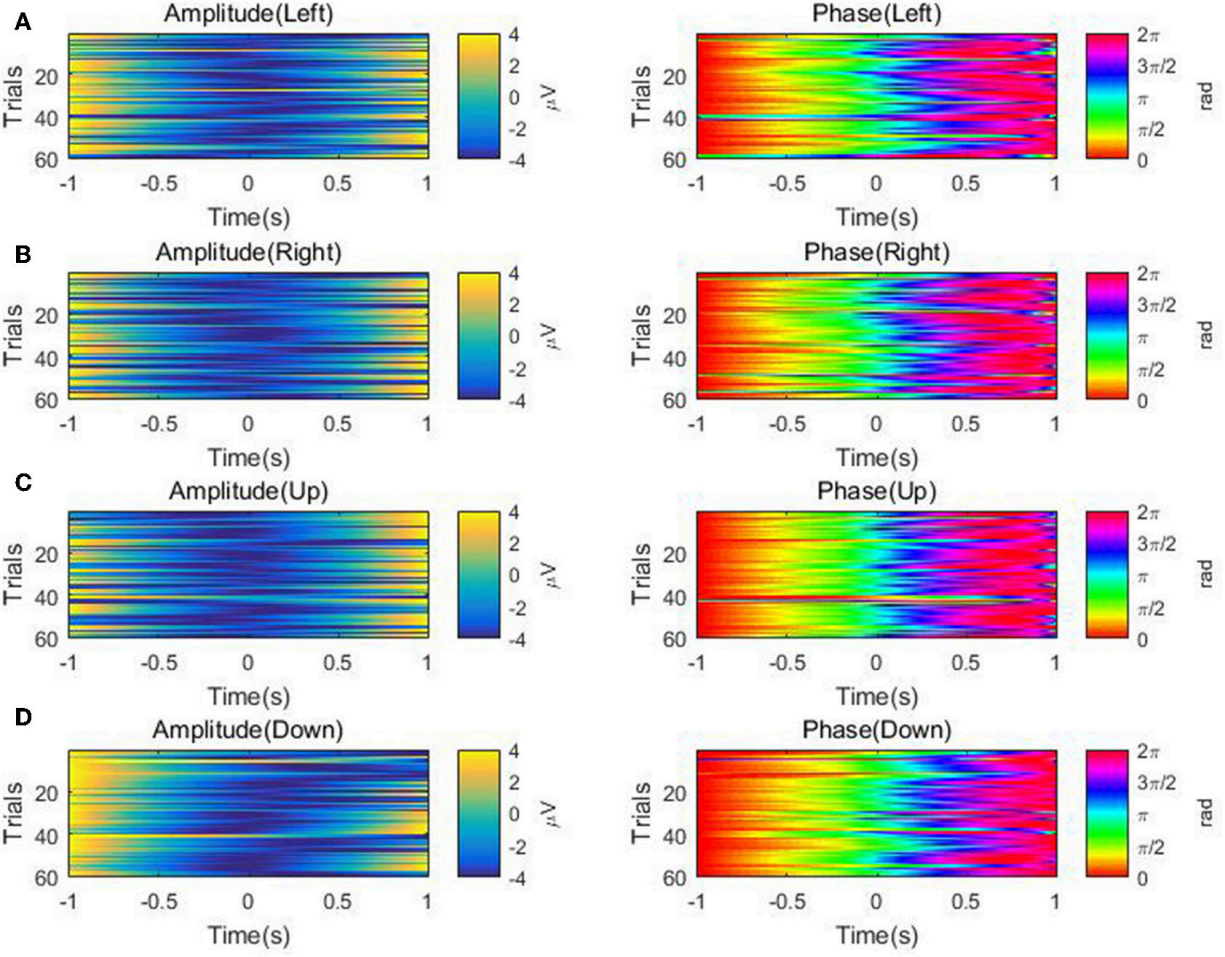
The temporal course of the low-delta amplitude and phase features across multiple trials over the channel FCz for the 2nd session of subject 5 along the four directions: **(A)** Left. **(B)** Right. **(C)** Up. **(D)** Down. Left panel: the amplitude feature. Right panel: the phase feature.

Furthermore, for each of the four directions, we visualize the temporal course of the low-delta EEG signal amplitude and phase features from channel FCz for a representative subject/session (i.e., the 2nd session of subject 5) across multiple trials in Figure 7. The amplitude temporal profiles are depicted in the top panels of Figures 7A–D. A decrease in amplitude before the onset of movement and an ascending trend in amplitude after the motion onset can be observed. The bottom panels of Figures 7A–D show that there is a synchronization of phase across multiple repetitions. After around 0.5 s prior to the movement onset, the bottom panels demonstrate a continuous increase in phase from π/2 to π. From the motion onset to the end of the hand movement around 0.5 s, there is an ascending trend in phase roughly from π to 3π/2. In a word, the temporal course of the low-delta EEG signal amplitude and phase features are generally in line with those reported in Sburlea et al. (2017), whose aim, however, is to detect the motion intention before the movement onset with the low-delta phase features. In our paper, such temporal profiles of low-delta EEG signals will be employed for the decoding of the continuous hand movement parameters after the movement onset.

### Decoding Performance

#### Evaluation Results Against the Decoding Models With the Low-Delta Amplitude Feature

**Figure 8:**
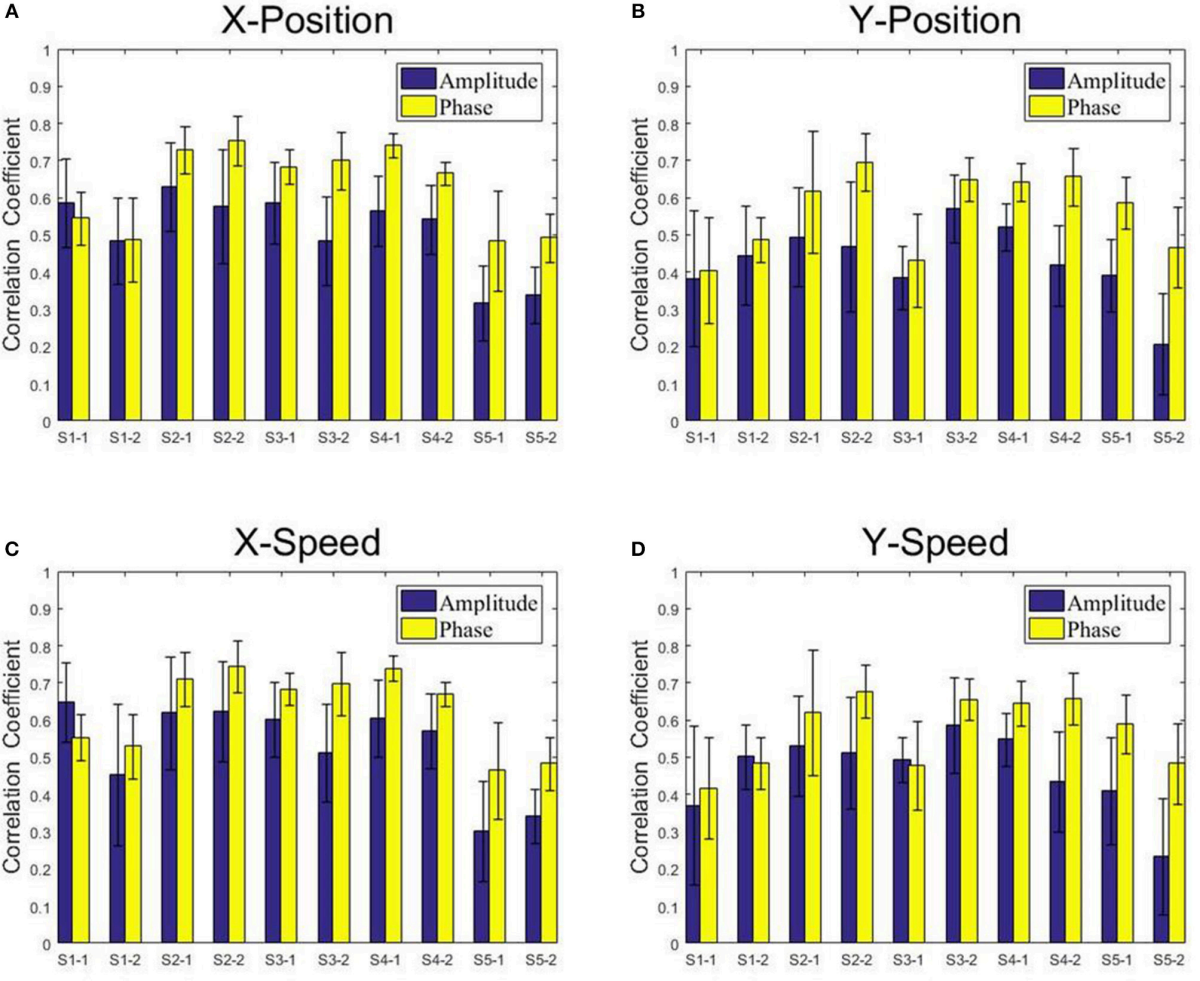
The correlation coefficients obtained from the multiple linear regression model with the low-delta EEG amplitude feature and the phase feature for each subject and each session. **(A)** X-Position. **(B)** Y-Position. **(C)** X-Speed. **(D)** Y-Speed.

**Figure 9:**
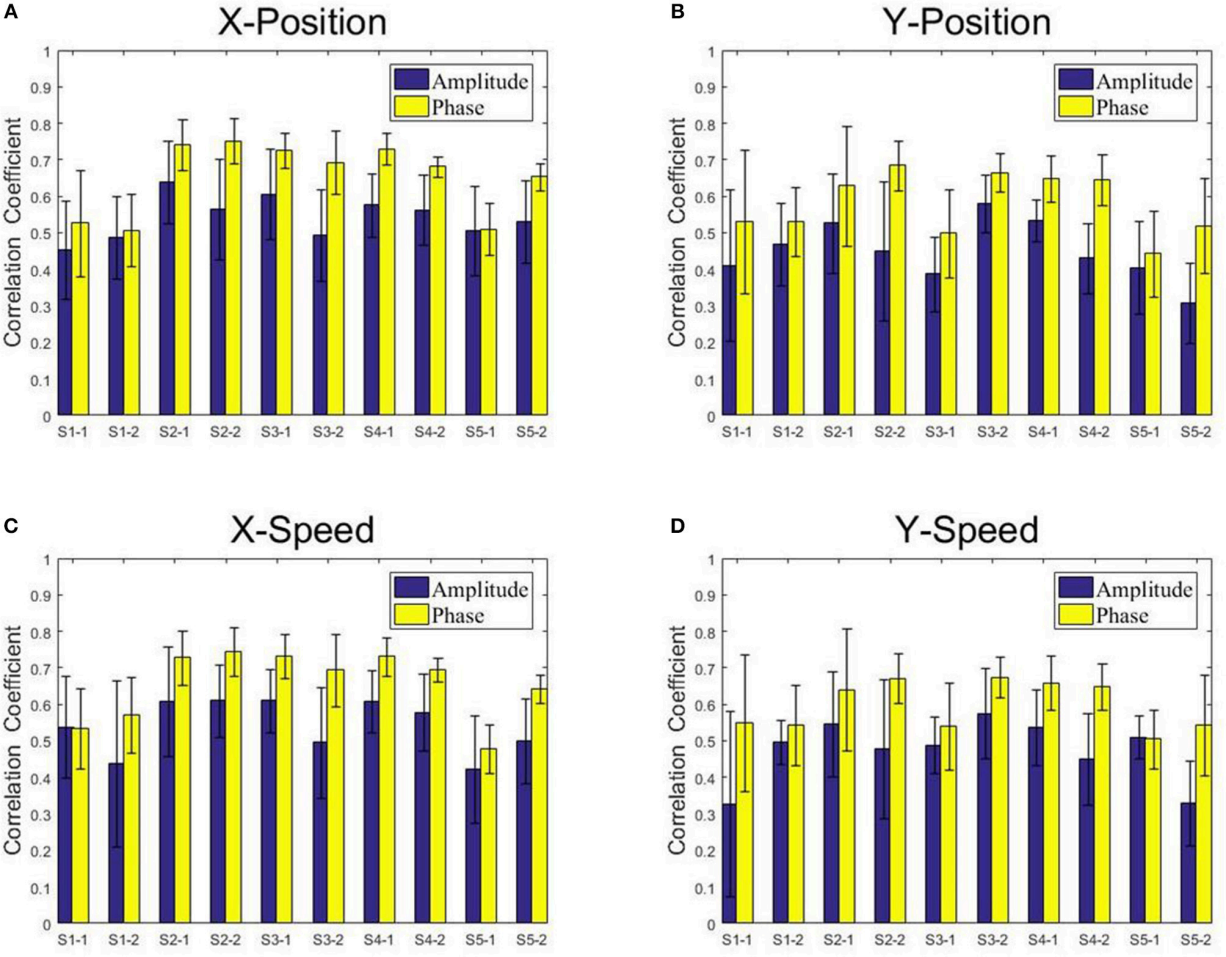
The correlation coefficients obtained from the Kalman filter model with the low-delta EEG amplitude feature and the phase feature for each subject and each session. **(A)** X-Position. **(B)** Y-Position. **(C)** X-Speed. **(D)** Y-Speed.

Figures 8, 9 present the center-out reaching hand movement kinematics (i.e., the positions along the X and Y axis: X-Position, Y-Position; the speed values along the X and Y axis: X-Speed and Y-Speed) six-fold cross validation decoding performance of the Phase model and the Amplitude model for each subject and each session using MLR and KF, respectively. Table 1 gives the six-fold cross validation decoding results across the subjects and sessions.

**Table 1:** The six-fold CV decoding results with low-delta EEG phase or amplitude feature.

		**X-Position**	**Y-Position**	**X-Speed**	**Y-Speed**
MLR	Phase	0.63 ± 0.11	0.56 ± 0.11	0.63 ± 0.11	0.57 ± 0.10
	Amplitude	0.51 ± 0.11	0.43 ± 0.10	0.53 ± 0.12	0.46 ± 0.10
	Signed-rank test *p*-value	0.0539	0.0018	0.034	0.003
KF	Phase	0.65 ± 0.10	0.58 ± 0.08	0.66 ± 0.10	0.60 ± 0.07
	Amplitude	0.54 ± 0.06	0.45 ± 0.08	0.54 ± 0.07	0.47 ± 0.08
	Signed-rank test *p*-value	0.0173	0.0091	0.0211	0.0036

**Figure 10:**
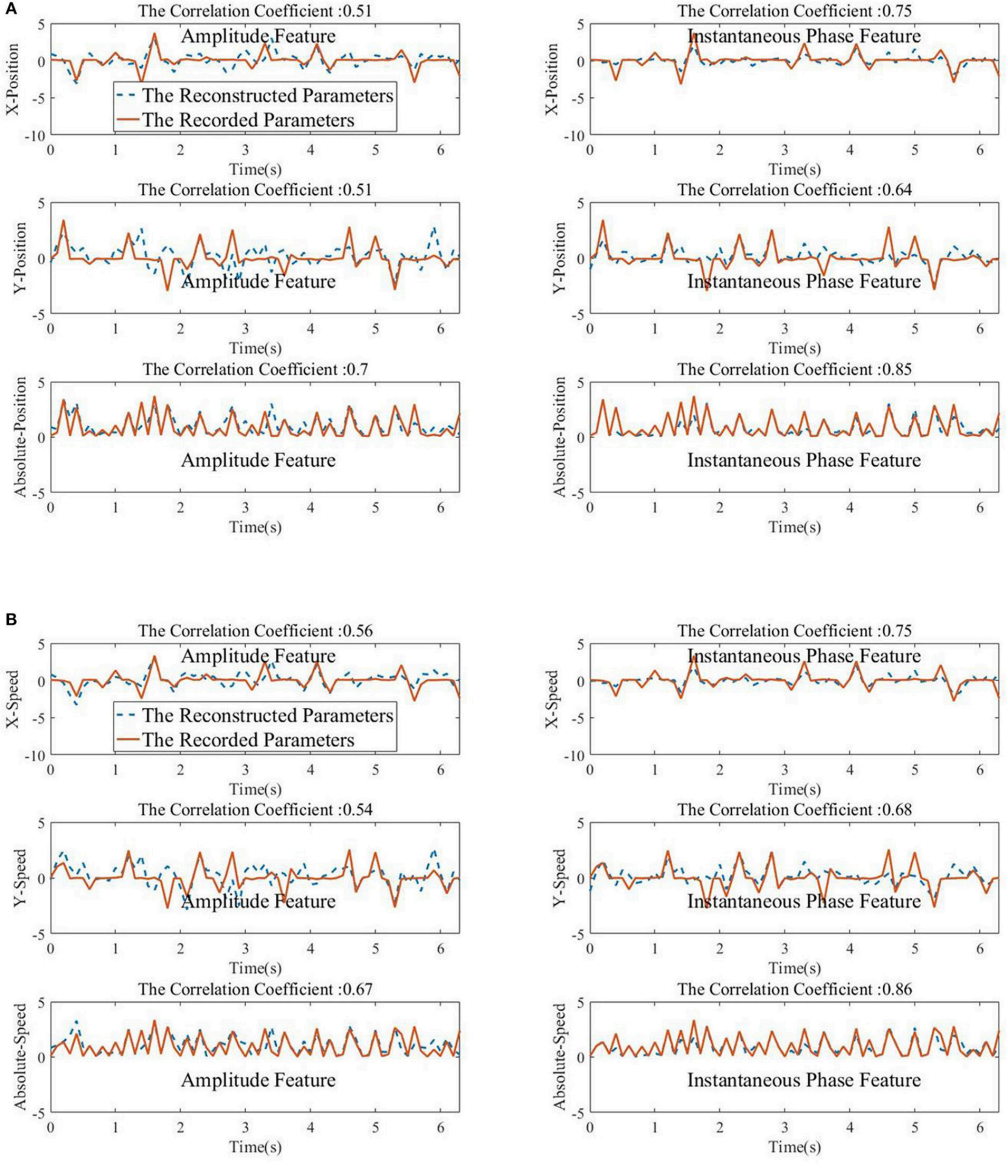
The Kalman filter reconstructed movements parameters (normalized for visualization) with the low-delta EEG amplitude feature (left column) and the phase feature (right column). The correlation coefficient between the predicted and the ground-truth parameters is given in the top of each subfigures. **(A)** Top panel: X-Position. Middle panel: Y-Position. Bottom panel: Absolute-Position. **(B)** Top panel: X-Speed. Middle panel: Y-Speed. Bottom panel: Absolute-Speed.

From Figures 8, 9, we find that the Phase model clearly proves to be substantially superior over the Amplitude model for almost all subjects and sessions, with both MLR and KF. Moreover, for the phase features, the correlation coefficients between actual and estimated position/speed are significantly higher than those for the amplitude features for almost all the subjects and sessions (except the X-Position decoding results with MLR, see Table 1). Representative hand movements reconstruction results for a single block and a single subject (the first block of the first session, subject 4) are shown in Figure 10, where the estimated absolute movement position and speed are also shown. It can be observed that the overlaps between the predicted and ground-truth parameters with the Phase model are more significant than those with the Amplitude model.

#### Evaluation Results Against the Decoding Models With Other-Band Power and Amplitude Features

Table 2 summarizes the configurations of the proposed decoder and the decoders with other-band power and amplitude features. The MLR and KF model based decoding CV performance metrics are given in Tables 3, 4, respectively. The best approach is underlined for each dataset.

**Table 2:** The configurations of the proposed low-delta EEG phase based decoder and the decoders based on power and amplitude features from other-band EEG.

**Decoder name**	**Configuration**	**Decoder name**	**Configuration**
Low δ-pha-MLR	Low δ (0.1–1 Hz) EEG phase based MLR	Low δ-pha-KF	Low δ (0.1–1 Hz) EEG phase based KF
High δ-pwr-MLR	High δ (1–4 Hz) EEG power based MLR	High δ-pwr-KF	High δ (1–4 Hz) EEG power based KF
θ-pwr-MLR	θ (4–8 Hz) EEG power based MLR	θ-pwr-KF	θ (4–8 Hz) EEG power based KF
μ-pwr-MLR	μ (8–12 Hz) EEG power based MLR	μ-pwr-KF	μ (8–12 Hz) EEG power based KF
β-pwr-MLR	β (12–30 Hz) EEG power based MLR	β-pwr-KF	β (12–30 Hz) EEG power based KF
High δ-amp-MLR	High δ (1–4 Hz) EEG amplitude based MLR	High δ-amp-KF	High δ (1–4 Hz) EEG amplitude based KF
θ-amp-MLR	θ (4–8 Hz) EEG amplitude based MLR	θ-amp-KF	θ (4–8 Hz) EEG amplitude based KF
μ-amp-MLR	μ (8–12 Hz) EEG amplitude based MLR	μ-amp-KF	μ (8–12 Hz) EEG amplitude based KF
β-amp-MLR	β (12–30 Hz) EEG amplitude based MLR	β-amp-KF	Beta (12–30 Hz) EEG amplitude based KF

**Table 3:** The six-fold CV position/speed reconstruction performance of the proposed low-delta EEG phase based decoder and the other-band EEG power and amplitude features based decoders using MLR.

**Dataset**	**low δ-pha-MLR**	**high δ-pwr-MLR**	**θ-pwr-MLR**	**μ-pwr-MLR**	**β-pwr-MLR**	**high δ-amp-MLR**	**θ-amp-MLR**	**μ-amp-MLR**	**β-amp-MLR**
S1-1	0.48/0.49	0.27/0.28	0.36/0.32	0.31/0.28	0.17/0.16	0.26/0.27	0.33/0.33	0.31/0.27	0.17/0.15
S1-2	0.49/0.51	0.28/0.37	0.34/0.34	0.23/0.26	0.12/0.12	0.27/0.31	0.35/0.37	0.21/0.26	0.11/0.13
S2-1	0.67/0.67	0.51/0.53	0.38/0.39	0.43/0.41	0.22/0.23	0.50/0.52	0.39/0.39	0.39/0.41	0.23/0.23
S2-2	0.72/0.71	0.46/0.52	0.45/0.45	0.42/0.45	0.28/0.27	0.47/0.52	0.42/0.45	0.41/0.44	0.24/0.27
S3-1	0.56/0.58	0.62/0.66	0.54/0.56	0.50/0.57	0.33/0.33	0.59/0.63	0.53/0.55	0.50/0.54	0.28/0.29
S3-2	0.67/0.68	0.53/0.65	0.53/0.56	0.47/0.48	0.25/0.25	0.56/0.59	0.54/0.55	0.47/0.45	0.22/0.21
S4-1	0.69/0.69	0.51/0.54	0.42/0.42	0.52/0.54	0.24/0.25	0.48/0.51	0.43/0.44	0.49/0.53	0.22/0.22
S4-2	0.66/0.66	0.42/0.44	0.50/0.53	0.48/0.46	0.41/0.42	0.43/0.46	0.53/0.53	0.46/0.49	0.41/0.41
S5-1	0.54/0.53	0.51/0.53	0.55/0.58	0.44/0.49	0.23/0.25	0.51/0.52	0.55/0.57	0.43/0.45	0.24/0.24
S5-2	0.48/0.48	0.42/0.41	0.34/0.38	0.41/0.44	0.27/0.26	0.41/0.41	0.35/0.36	0.38/0.41	0.23/0.24
Mean	0.60/0.60	0.45/0.49	0.44/0.45	0.42/0.44	0.25/0.25	0.45/0.48	0.44/0.45	0.40/0.43	0.23/0.24

*The best approach is underlined for each dataset*.

**Table 4:** The six-fold CV position/speed reconstruction performance of the proposed low-delta EEG phase based decoder and the other-band EEG power and amplitude features based decoders using KF.

**Dataset**	**low δ-pha-KF**	**high δ-pwr-KF**	**θ-pwr-KF**	**μ-pwr-KF**	**β-pwr-KF**	**high δ-amp-KF**	**θ-amp-KF**	**μ-amp-KF**	**β-amp-KF**
S1-1	0.53/0.54	0.32/0.23	0.43/0.39	0.33/0.30	0.17/0.20	0.28/0.23	0.40/0.36	0.31/0.28	0.18/0.17
S1-2	0.52/0.55	0.27/0.34	0.38/0.42	0.26/0.28	0.09/0.13	0.30/0.33	0.36/0.41	0.22/0.28	0.11/0.13
S2-1	0.69/0.68	0.55/0.58	0.43/0.39	0.34/0.40	0.27/0.30	0.54/0.55	0.42/0.40	0.36/0.38	0.28/0.28
S2-2	0.72/0.71	0.38/0.41	0.45/0.49	0.43/0.45	0.29/0.32	0.38/0.43	0.44/0.48	0.41/0.44	0.28/0.30
S3-1	0.61/0.64	0.53/0.58	0.55/0.58	0.50/0.50	0.32/0.33	0.56/0.58	0.53/0.54	0.47/0.49	0.30/0.32
S3-2	0.68/0.68	0.53/0.57	0.59/0.58	0.50/0.51	0.26/0.26	0.52/0.53	0.55/0.57	0.50/0.50	0.26/0.23
S4-1	0.69/0.69	0.44/0.47	0.41/0.48	0.54/0.55	0.26/0.22	0.44/0.47	0.43/0.45	0.51/0.53	0.24/0.24
S4-2	0.66/0.67	0.42/0.42	0.58/0.51	0.48/0.50	0.42/0.45	0.42/0.43	0.55/0.55	0.46/0.47	0.43/0.44
S5-1	0.48/0.49	0.46/0.51	0.53/0.63	0.42/0.55	0.22/0.24	0.45/0.49	0.54/0.61	0.41/0.50	0.19/0.24
S5-2	0.59/0.59	0.46/0.47	0.48/0.43	0.55/0.56	0.28/0.27	0.47/0.48	0.44/0.44	0.51/0.53	0.25/0.26
Mean	0.62 /0.63	0.44/0.46	0.49/0.49	0.44/0.46	0.26/0.27	0.43/0.45	0.47/0.48	0.42/0.44	0.25/0.26

*The best approach is underlined for each dataset*.

With the MLR model, the proposed decoder is the best one on 8 out of the 10 datasets for both the position and speed reconstruction. Similarly, with the KF model, the proposed approach wins on 9 out of the 10 datasets for both the position and speed reconstruction. Moreover, all the Jarque-Bera test results have demonstrated the failure to reject the null hypothesis that the CV performance comes from a normal distribution at the 0.05 significance level. Pairwise comparisons between our proposed method and each of the rest indeed shows statistically significant differences (*p* < 0.05) for all the results presented in Tables 3, 4. In contrast, the mu-band and beta-band power feature based decoders (μ/β-pwr-MLR/KF) fail to show advantageous performance over the rest counterparts. In addition, μ-amp/pwr-MLR/KF, high δ-amp/pwr-MLR/KF, and θ-amp/pwr-MLR/KF produced similar performance, but none of the pairwise performance comparisons among them is statistically significant (*P* > 0.05).

#### Evaluation Results Against the Chance-Level Decoding Models

The chance-level results are considerably worse than the authentic models, and the performance difference is statistically significant (see Table 5). This indicates that the proposed Phase decoding model has indeed employed the neural correlates of the hand movement kinematics and the decoding performance obtained not by chance. Taken together, these results clearly suggest that the phase is the major carrier of the low-delta EEG movement-related information.

**Table 5:** Reconstruction results by chance-level models with the low-delta EEG phase feature.

		**X-Position**	**Y-Position**	**X-Speed**	**Y-Speed**
Phase-MLR	Authentic model	0.63 ± 0.11	0.56 ± 0.11	0.63 ± 0.11	0.57 ± 0.10
	Chance-level model	0.23 ± 0.06	0.22 ± 0.06	0.24 ± 0.07	0.24 ± 0.06
	Rank-sum test *p*-value	<< 1e-5	<< 1e-5	<< 1e-5	<< 1e-5
Phase-KF	Authentic model	0.65 ± 0.10	0.58 ± 0.08	0.66 ± 0.10	0.60 ± 0.07
	Chance-level model	0.21 ± 0.05	0.20 ± 0.06	0.22 ± 0.05	0.23 ± 0.06
	Rank-sum test *p*-value	<< 1e-5	<< 1e-5	<< 1e-5	<< 1e-5

## Discussion

### Role of Low-Delta EEG Phase Features in Decoding Kinematics for Motor Control

In the domain of sensory processing, it has been recently suggested that the instantaneous phase patterns of the oscillatory activity in theta and alpha bands contain neural correlates of the brain response to visual/auditory stimuli (Ng et al., 2013; Wang et al., 2018). For cognition, there is also accumulating evidence that the phase patterns of slow EEG oscillation can be informative about the cognitive task being performed (Höhne et al., 2016; Barry et al., 2018). Moreover, these works suggest that the sensory or cognitive information carried by low-frequency EEG signals is richer and greater in their precise temporal dynamics (phase) compared with their amplitude. These studies have demonstrated the phase of low- frequency EEG signals as an effective neural correlate for sensory and cognition processing.

In the motor control domain, most studies have explored the EEG amplitude or power representation of the movement-related neural correlates (Miao et al., 2016; Robinson and Vinod, 2016; Zeng and Song, 2016)(Miao et al., 2016; Robinson and Vinod, 2016; Zeng and Song, 2016). In the past decades, the connectivity metric among brain areas has been gradually investigated mainly using the phase patterns of the mu band, which have demonstrated to contain the motor imagery information (Hamedi et al., 2016). Moreover, recent studies have reported that the instantaneous phase representation of the low-delta (0.1–1 Hz) band not only yields higher motion detection accuracy before the movement onset, but also detects the motion intention much earlier than the amplitude (Sburlea et al., 2017; Zeng et al., 2017b). Based on these findings, the starting point of our study is to investigate whether the continuous kinematic information after the movement onset could be decoded from the phase representation of the 0.1–1 Hz delta band, and whether it is superior to the amplitude representation of the same signal. Our neurophysiological analysis results indicate that on representative channels, the instantaneous phase representation of the lower delta band shows higher signal-to-noise ratio and stronger modulation by the movement tasks than the amplitude one. Such characteristics of phase have led to higher decoding accuracy of the kinematic information after the movement onset compared to the slow-oscillation amplitude based decoders. Taken together, these results clearly suggest that the motor control imprints more on the precise dynamics than on the amplitude of the slow rhythmic brain activity.

### Toward Understanding the Strength of Methods and Paradigms Used

In section Decoding Performance, we have shown quantitative evidences suggesting that the instantaneous phase of the low-delta EEG constitutes a powerful representation for reconstructing the hand movement parameters. In this section, we shall provide closer analysis and visualization to further understanding the strength of the low-delta EEG signal phase representation and the movement paradigm.

First, it is necessary to point out an important characteristic of the center-out reaching movement paradigm in our two-session experiments. Namely, all the subjects completed the self-paced center-out reaching movement within 0.5 s, and the minimum movement duration was 0.2 s. Next, from Figure 7, we can observe that the low-delta EEG signal phase during the hand movement interval [0, 0.5] s is distributed roughly in the interval [π, 3π/2], rather than the interval [0, 2π] where a general phasic circular variable is distributed and possibly with sharp discontinuities as well. In addition, the extremities of such an interval [π, 3π/2] are not close to each other in the polar coordinates. Moreover, the phase value varies continuously along the motor task across trials. Such a phase representation is further investigated on several representative channels (see Figures 4, 6). Here, it is necessary to point out that such representative channels are particularly screened over the frontal and parietal cortices, since such two brain regions have been extensively reported to contain necessary information for decoding planning and execution of reaching movements in the literature (Lew et al., 2014; Robinson et al., 2015). On such representative channels, the phase representation has demonstrated similar sharp transit around the motion onset as the corresponding kinematic parameters (see Figures 4, 6), and Figure 5 further reveals that the phase representation strongly correlated with the corresponding kinematic parameters. Such observations, to some extent, support our conjecture that the hand movement kinematic parameters during the movement tasks in our experiment paradigm can be obtained from the linear decoding models with the phase representation distributed in [π, 3π/2]. Therefore, instead of treating it as a circular variable, we simply applied the z-score procedure as well as the linear decoding model on the continuous phase variable during the motor task period. According to the decoding results, the feasibility and effectiveness of our approach have been validated.

### Comparison of Executed 2D Hand Movement Decoding Performance of Previous Methods With EEG

The EEG signals have been investigated in many studies (Lv et al., 2010; Heger et al., 2012; Agashe et al., 2015; Robinson et al., 2015) for the decoding of the hand/finger/elbow kinematic parameters during 2D/3D center-out movement, natural grasping, filling a glass of water, and etc. These studies have employed either the EEG amplitude or power representation from some particular band, whereas the phase representation in the low-frequency band EEG has not yet been explored for the continuous kinematic parameters decoding after the movement onset.

In previous studies using low-delta band amplitude based linear decoding models and similar 2D executed hand movement tasks as ours, Lv et al. (2010) has achieved an average CC around 0.46 and Robinson et al. (2015) has reported values around 0.57. Our implemented low-delta band amplitude based model resulted in the best decoding accuracy (CC = 0.54 ± 0.06, mean ± standard deviation) for position in X-axis with Kalman filter, and are thus consistent with the previous low-delta amplitude based decoding investigations. By contrast, with the proposed delta band instantaneous phase representation in our study, the Kalman filter decoding accuracy for position in X-axis can be improved up to CC = 0.65 ± 0.10 (mean ± standard deviation), with statistical significance. In addition, although the mu or beta bandpower features have been found to be effective for estimating the 3D imagined upper limb continuous movements in Korik et al. (2016, 2018), such representations and the amplitude features in mu or beta band are demonstrated to be less effective than the low-delta phase feature for the decoding of continuous kinematic parameters in our 2D executed movement tasks.

### Limitations and Future Work

For the decoding models in our study, we set the time lag to be 11 according to the suggestions in Bradberry et al. (2009, 2010) and Robinson et al. (2015)(Bradberry et al., 2009, 2010; Robinson et al., 2015). Optimization of such a parameter through cross-validation may further lead to improved performance. Besides, since the average decoding accuracy has been greatly improved from 0.54 (amplitude) to 0.66 (phase) with statistical significance using all the 33 channels, we have not performed the channel selection procedure in our approach. It may certainly result in enhanced performance by conducting the channel selection.

As in most studies on kinematics decoding with EEG signals, only the linear decoding models such as MLR and KF were employed in our paper. In the future, we will further investigate whether the phase based non-linear decoding model would improve the performance of the linear ones. Moreover, the low-delta phase feature as well as the amplitude/power features from other bands, were employed separately for reconstructing the center-out reaching hand movement trajectories. They may reflect different aspects of the EEG signals during the motor control process. For instance, the low-delta phase describes the temporal dynamics of the movement-related brain activities, while the low-delta amplitude and mu band power characterize the intensity information. For the future work, we will try to build advanced decoding models with these three representations combined together in order to fully utilize the complementary information in them. Last but not least, we have only studied the trajectory reconstructing problem for an executed 2D center-out reaching task in the current work. To realize the futuristic neuroprosthesis control for paralyzed patients using non-invasive EEG, it is desirable to investigate the trajectory reconstructing problem for the imagined movements. Although we have shown that the low-delta EEG phase has yielded better decoding accuracy than the compared features, for a 2D hand center-out reaching movement task, it is unknown whether such a feature could still provide the best performance among these investigated features in the paper, for decoding the imagined 2D or 3D continuous movements. As a future work, this study will be further extended to systematically examine such an issue.

## Conclusion

In this paper, we have investigated the phase representation of low-delta EEG signals in decoding 2D center-out reaching hand movement parameters. The neurophysiological analysis has shown that on representative channels over the cortices that are known to encode the execution information of reaching movements, the low-delta EEG instantaneous phase representation has a higher signal-to-noise ratio and stronger modulation by the movement tasks, compared to its amplitude representation. Next, we have compared the performance of the low-delta EEG phase based liner decoding model and its amplitude based one, and the results demonstrate that the decoding accuracy based on amplitude is substantially lower than that based on phase, with statistical significance. Furthermore, the experimental results have also validated the advantages of the low-delta phase feature over the time-resolved power and amplitude series from other frequency bands for such a 2D executed movement task. Our findings clearly pinpoint that the low-delta EEG phase contains comprehensive movement related information, demonstrating its potential for the futuristic fine neuroprosthesis control with continuous movement parameters reconstructed from non-invasively recorded scalp EEG.

## Ethics Statement

This study was carried out in accordance with the recommendations of the Ethics Committee of Southeast University with written informed consent from all subjects. All subjects gave written informed consent in accordance with the Declaration of Helsinki. The protocol was approved by the Ethics Committee of Southeast University.

## Author Contributions

HZ and YS designed the study, analyzed the data, and wrote the manuscript. CW and GX set up the experiment platform. BX and HL performed the experiment. AS, HL, BX, and CH were involved in critical revision of the manuscript. All authors read and approved the final manuscript.

### Conflict of Interest Statement

The authors declare that the research was conducted in the absence of any commercial or financial relationships that could be construed as a potential conflict of interest.

## Footnotes

^1^F3, F1, FZ, F2, F4, FC5, FC3, FC1, FCZ, FC2, FC4, FC6, C5, C3, C1, CZ, C2, C4, C6, CP5, CP3, CP1, CPZ, CP2, CP4, CP6, P5, P3, P1, PZ, P2, P4, P6.

^2^The shortest duration of the movement was 0.2 s in our experiment, therefore, the effect size for each type of feature was analyzed only in the movement sub-interval [0, 0.2] s.
